# Evaluating the Impacts of Patient Engagement on Health Services Research Teams: Lessons from the Veteran Consulting Network

**DOI:** 10.1007/s11606-021-06987-z

**Published:** 2022-03-29

**Authors:** Vanessa L. Merker, Justeen K. Hyde, Abigail Herbst, Amanda K. Solch, David C. Mohr, Lauren Gaj, Kelly Dvorin, Eileen M. Dryden

**Affiliations:** 1 Center for Healthcare Organization and Implementation Research (CHOIR), VA Bedford Healthcare System, Bedford, MA USA; 2 Department of Neurology and Cancer Center, Massachusetts General Hospital, MA, Boston, USA; 3 Center of Helathcare Organization and Implementation Research (CHOIR), VA Boston Healthcare System, Boston, USA

**Keywords:** stakeholder engagement, qualitative research, health services research, veterans, program evaluation

## Abstract

**Background:**

Despite increasing commitment to patient engagement in research, evaluation of the impact of these efforts on research processes, products, and teams is limited.

**Objective:**

To explore the impacts of engaging patients as consultants to research studies by examining the experiences, impacts, and lessons learned from a program facilitating patient engagement at a Veterans Health Administration research center.

**Design:**

We developed a logic model to articulate the activities being implemented to support patient engagement and their anticipated outcomes. Then, we conducted qualitative, semi-structured interviews with participants in the local Veteran Consulting Network to qualitatively explore these outcomes.

**Participants:**

Twelve researchers and eleven Veteran patients with experience working on at least one grant or funded study.

**Approach:**

Interview transcripts were inductively coded using a consensus-based approach. Findings were synthesized using framework analysis and mapped back onto our logic model of expected patient engagement impacts.

**Key Results:**

Patient engagement improved the perceived quality and relevance of research studies as patient consultants challenged researchers’ assumptions about patient populations and clinical contexts and gave feedback that helped improve the feasibility of proposed grants, readability of study materials, comprehensiveness of study assessments, and cultural sensitivity and relevance of interventions. Patient engagement also had personal benefits to researchers and patients. Researchers reported improved communication skills and higher job satisfaction. Patients reported a sense of purpose and satisfaction from their work with greater awareness of and appreciation for research.

**Conclusions:**

Engaging patients in research can have multiple benefits to the people and work involved. Our evaluation process can serve as a template for other organizations to plan for and assess the impact of their own patient engagement programs. Creating logic models and updating them based on feedback from program users make engagement goals explicit, help verify expected mechanisms to achieve impact, and facilitate organizational learning.

**Supplementary Information:**

The online version contains supplementary material available at 10.1007/s11606-021-06987-z.

## INTRODUCTION

Engagement of patients as consultants in research (“patient engagement”) is gaining recognition as a practical, ethical, and political imperative for health research.^1–4^ Emerging evidence suggests patient engagement leads to more meaningful, relevant, and actionable research findings that will ultimately improve population health.^5–7^ For this reason, research funders worldwide are increasingly encouraging or even requiring patient engagement in research studies.^8–11^ Patients may consult on a wide variety of research tasks, including framing research questions, selecting outcome measures, designing study recruitment plans, and facilitating the dissemination of study findings.^5, 12^ However, implementation of patient engagement efforts varies widely in regards to the types and timing of engagement activities used and patients’ level of power and decision-making authority.^13, 14^

Measuring and comparing the impacts of different patient engagement models can be difficult. Literature has begun to connect patients’ contributions to downstream effects^5^ but it is often uncertain how patient engagement affects the research process or research outcomes.^15^ While evaluation tools are available to track researchers’ assessment of the context, process, or outcomes of patient engagement, many are not based on explicit conceptual frameworks and/or lack psychometric validation.^16, 17^ Additionally, conceptual models defining impacts of patient engagement often ignore personal benefits for patients and the researchers they work with.^18^ Given the desire to foster equal partnerships within engaged research teams,^19^ it is imperative to judge patient engagement efforts not only by how they serve researchers’ needs, but also by whether they fulfill patients’ interests and goals.^20^

**Figure 1:**
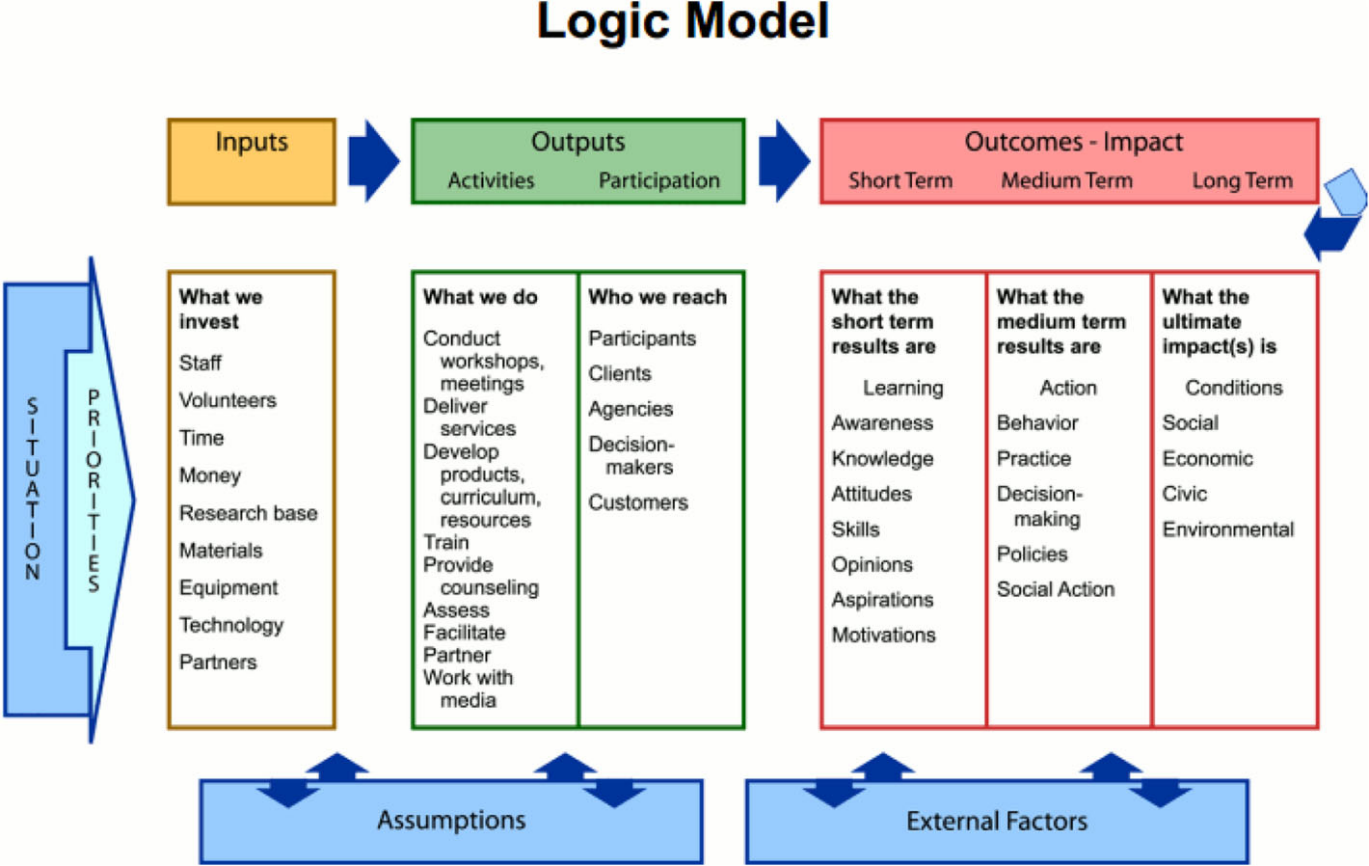
Logic model template. Example template for a logic model, including potential categories of inputs, outputs, and outcomes users may want to include in a logic model. Reprinted from Taylor-Powell, Jones, and Henert (2003) with permission from the University of Wisconsin Division of Extension.^22^

Given the current limitations in quantitative assessment of patient engagement impacts, qualitative evaluations guided by robust conceptual models remain a valuable tool to understand the impacts of patient engagement programs.^21^ Logic models are commonly used in program evaluation to visually depict the proposed mechanisms by which programs achieve desired changes (Fig. 1).^22–24^ Creating logic models of patient engagement can help program organizers articulate how planned engagement activities are expected to achieve program objectives^25^ and help program evaluators identify and categorize appropriate metrics to measure patient engagement.^26, 27^ By highlighting the short-term outcomes required to achieve long-term impact, logic models can help program evaluators focus on the specific objectives and timeframe relevant to each hospital system’s local patient engagement efforts. We present an example of how to use this approach to demonstrate the impact of patient engagement on research studies and the patients and researchers who partner together to conduct them.

## METHODS

### Setting

In an effort to make the research it funds more relevant to the patients it serves, the US Department of Veterans Affairs (VA) encourages and financially supports structured patient engagement opportunities for Veterans.^25, 28^ Many VA research centers have developed Veteran Engagement Groups, in which a standing advisory board of Veterans provide ad-hoc feedback on multiple research projects.^28^ Our research center—the Center for Healthcare Organization and Implementation Research (CHOIR)—developed an alternative model in which Veteran patients are matched to specific research teams to work together longitudinally on a single project. This unique model, referred to as the Veteran Consulting Network (VCN), was designed to efficiently facilitate a more collaborative approach to patient engagement across multiple health services research teams.

Patients are recruited to the VCN from two CHOIR-affiliated VA hospitals and related outpatient clinics (located in eastern Massachusetts). A program coordinator screens interested patients and enters information about their areas of interest and demographic characteristics into a database. The program coordinator then uses this database to match research teams, upon their request, with potential patient consultants. Researchers meet with patients to decide if the collaboration is mutually agreeable, determine the patients’ specific responsibilities, and provide all necessary training to enable patients’ participation. All patient consultants are compensated $25 per hour, with funds budgeted from research grants; consultations on unfunded proposals are subsidized by CHOIR central funds. As of July 2020, at least 17 patients (out of 53 potential volunteers) had consulted on 29 different grants with 23 different principal investigators; patients could consult on multiple projects and each project could have one or multiple patient consultants. Patient consultants are valued for adding their personal insights and contributions to the research team and are not expected to necessarily represent all VA patients’ views.^29, 30^

### Development of Veteran Engagement Logic Model

In order to evaluate the impact of patient engagement in our organization, a logic model was developed to visually articulate the overall goals of the VCN, key activities to support patient engagement, and the anticipated outcomes of such engagement for patients, researchers, and the research center. These outcomes were selected based on the review of literature on patient-engaged research and early experiences with VCN participants. The logic model went through several iterations, with input from patients, researchers, and CHOIR’s leadership. Compared to more static conceptual model espousing a set theory to be tested, this logic model is considered a “living document” and serves as an organizing guide for evaluation activities. Using the logic model was particularly helpful for this early evaluation by focusing our inquiry on the short-term outcomes most relevant to our objectives, and fostering critical questioning of whether expected short-term outcomes were necessary to drive longer term changes.

### Participants and Data Collection

**Figure 2:**
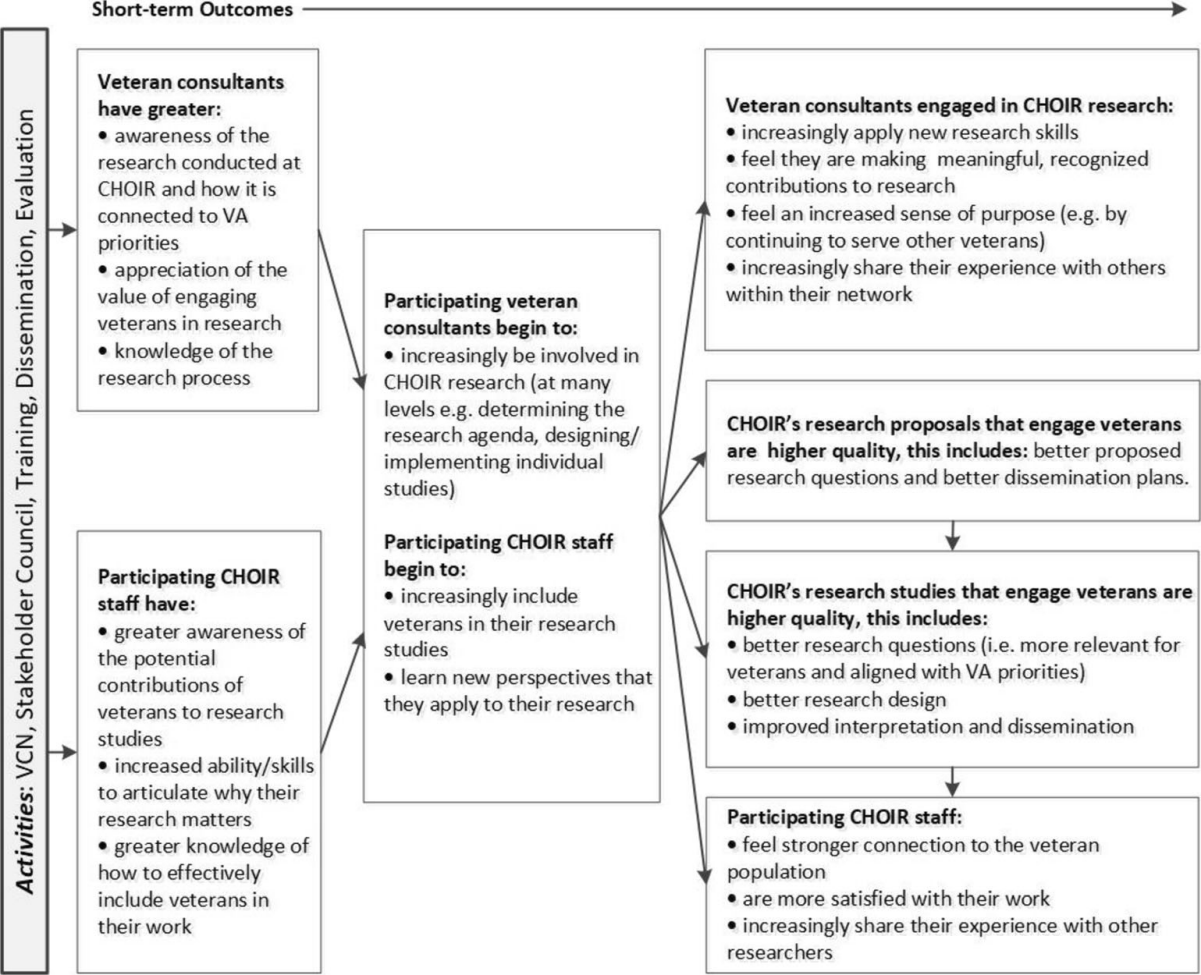
Logic model of expected short-term outcomes of veteran engagement. Anticipated short-term outcomes from the Veteran Consulting Network, including hypothesized outcomes for patient consultants, researchers, and research proposals/studies. The figure represents a subset of the original logic model for veteran engagement at CHOIR, focusing on the outcomes which guided the present evaluation.

As part of a quality improvement initiative, qualitative interviews were conducted with patient consultants and researchers involved in the VCN to assess the extent to which the program is generating anticipated short-term outcomes outlined in the logic model (Fig. 2). Participants were purposively sampled to identify those most experienced with the VCN (i.e., long-term involvement over the lifecycle of a patient-engaged project or involved in multiple patient-engaged projects). Interviews were conducted in-person or over the phone by the first author using semi-structured interview guides (Appendix 1). Interviews began with a wide, open-ended focus on each participant’s experience on a patient-engaged research team, and then specific probes were used to explore the hypothesized impacts of patient engagement. Interviews were audio-recorded with participant consent and transcribed verbatim; for two participants without recordings, detailed interview notes were collected instead. Patients received $25 for their participation.

### Data Analysis

We analyzed interviews using Framework Analysis—a systematic approach involving familiarization with interviews, initial coding, developing and applying an analytical framework, charting data into matrices, and interpreting the data.^31, 32^ Specifically, the coding team (five health services researchers) reviewed initial transcripts line-by-line to identify codes that represented the meaning of the text. These inductive codes were combined with deductive codes derived from our original logic model (Fig. 2) to create an analytical framework, which was iteratively refined as the team came to a consensus on code definitions and application of codes in the initial transcripts. The team independently coded the remaining transcripts, meeting regularly to discuss any questions of coding application and to enhance coding consistency across team members.^33^ Final consensus codes were recorded in NVivo 12. Data encompassed by each code were systematically reviewed and categorized using matrices that included names and definitions of common themes and sub-themes, participants IDs, and representative quotes. With input from additional CHOIR researchers and a Veteran consultant (none of whom participated in interviews), we finalized key themes via collaborative discussion, compared our results to our original logic model, and revised the model to incorporate new findings.

## Results

Eleven patient consultants and twelve researchers (including 7 principal investigators and 5 staff members) were interviewed between August and October 2019. Interviewee demographics were broadly reflective of the population of patients and researchers at our center (Table 1) and highlight the unique experiences Veteran patients added to predominantly non-Veteran research teams. Interviewees largely valued patient engagement in research and highlighted multiple impacts of adding patients’ insights and experiences to the research team (Table 2).

**Table 1:** Study Participant Demographics

	Patient consultants (*n*=11)	Researchers (*n*=12)
Gender		
Male	9 (82%)	3 (25%)
Female	2 (18%)	9 (75%)
Race		
White	8 (73%)	8 (67%)
Black	2 (18%)	0 (0%)
Asian	1 (9%)	3 (25%)
Biracial/multiracial	0 (0%)	1 (8%)
US Veteran		
Yes	100 (100%)	1 (8%)
No	0 (0%)	11 (92%)

**Table 2:** Short-term outcomes of veteran consulting network

	Patient Consultants	Researchers	Research Products
Confirmed Outcomes	• Greater awareness of ongoing research and its’ connection to health system priorities •Appreciate value of patient engagement •Greater knowledge of research process •Make meaningful, recognized contributions to research •Feel an increased sense of purpose	•Appreciate value of patient engagement •Greater knowledge of how to effectively engage patient consultants •Increased ability to communicate why their research matters •Learn new perspectives to apply to research •Feel stronger connections to patient population •More satisfied with their work	•Higher quality grant proposals with more relevant research questions and better dissemination plans •Research studies with more relevant research questions
New Outcomes	•Increased social connections with researchers and other patients	•Greater understanding of patients’ experiences •Improved ability to communicate about research methods	•More feasible and acceptable interventions •More feasible and culturally appropriate study instruments/ tools
Unobserved Outcomes	•Apply new research skills •Discuss engagement experience within their social network	•Discuss engagement experience within their social network	

### Impact on the Quality and Relevance of Research

Researchers reported that patient engagement provides the following benefits: (1) opportunities to confront assumptions about patient populations and clinical care contexts that influence research; (2) improvements in the quality of research questions, grant submissions, and study materials; and (3) improvements in intervention designs.

#### Challenging Underlying Assumptions that Influence Research

Patient engagement provided researchers with new perspectives that challenged assumptions underpinning research questions and study designs. Consulting with patients gave researchers more insight into the diversity of Veterans’ experiences in the military and their lives post-service. As one researcher noted:

I try to be more cautious in research teams when we talk about the Veteran experience and when we’re trying to think about how our research applies to Veterans. I think part of what it does is it makes us realize that each Veteran can be quite different… (Investigator 02)

Consulting regularly with patients also helped researchers see their “*blind spots*” regarding intervention designs and develop a new appreciation for patients’ real-world challenges. One team was developing an intervention using the VA’s online patient portal (My HealtheVet). Their assumptions about the portal’s ease of use had influenced their early decisions about how to deploy the planned intervention, until they consulted with a patient:

[One patient] logged into his My HealtheVet account with us. [And we looked at] how he could maneuver through the website. So I think that was really an eye-opening experience for us. Just how challenging it can be to navigate through these things and how long it can take. So that really helped us better design the training and made us more realistic about how much are you gonna’ squeeze into an hour. (Staff 01)

Overall, patient engagement was helpful in challenging researchers’ assumptions regarding the structures, processes, relationships, or resources that are generally available within clinical contexts. This was particularly true for non-clinician researchers who interacted less with patients:

I’m not a clinician, so I don’t see patients, right? So I think there’s a bigger detachment for me. I do this research but I don’t know what are patients thinking or what their perspective is. So I think having that conversation with her kind of brought to the forefront for me what other problems patients have (Investigator 01)

This researcher, studying care for post-menopausal female Veterans, described how a patient consultant encouraged her not to assume that physicians were having conversations about this life transition with patients. This led the researcher to modify her research question and methods to better account for variability in menopause screening and documentation.

#### Improvements to Grant Submissions, Research Studies, and Study Instruments

Researchers highlighted a range of improvements to research products resulting from patient engagement. In some cases, patient consultants identified issues or concerns with research questions or approaches that grant reviewers may also have, allowing researchers to proactively address them in the grant.

[We asked consultants,] ‘Do people think that kind of [potentially sensitive] question would be answered [by study participants]?’ You know, it was valuable to see there were mixed views on that. It wasn’t black and white… So I think it prepared me for the kind of pushback I might get from reviewers and it then led, I think some of that led to some focus group work I did prior to the third [grant] submission. (Investigator 02)

As this investigator noted, conversations with patient consultants allowed them to flesh out ideas in greater detail before grant submission and identify needs for additional pilot work to communicate the feasibility and acceptability of research designs.

Patient engagement also highlighted the need to include additional survey or interview questions to fully capture the research domain of interest and/or provide important context to patient responses. For example, one researcher who engaged a patient from the beginning of the grant attributed one of the most significant findings of the study to recommendations that the patient consultant made to the data collection instrument.

…we asked [name], you know, what do you think we should be asking Veterans? What’s important to them? And he came up with a question that was like, ‘How well were your needs met at your primary care appointment’. He said that was the most important thing. Like nothing else matters but did I get what I came here for. So we all thought that was really great and so we added that on to the [survey] questions and it ended up being that it was well correlated with the [primary measure] that we had. (Staff03)

Patient consultants also improved study instruments (e.g., interview guides, and surveys) in terms of how the study purpose was explained, how questions were phrased, and overall formatting. Some teams had significant involvement of patient consultants in “getting their perspective on the length and the tone, making sure that all the key components of what they experienced as patients were reflected” (Investigator 07). These reviews resulted in significant redesigns to improve the flow and ease of completion.

#### Improvement to Interventions

Overall, working with patient consultants representing an intervention’s target population raised awareness of important end-user issues and concerns. In some cases, patient consultants provided feedback to make intervention content more culturally sensitive and respectful. In others, patient consultants highlighted challenges with intervention formats and made suggestions for how to improve ease and acceptability of interventions by modifying or adding content.

[The patient] suggested instead of just having a training guide on paper, you know, he told us that sometimes he gets tired reading. So what about creating some videos that patients could watch? So we took his advice and we created some videos…if it weren’t for the fact that he came with these vision issues [related to the health condition for which the intervention was being developed], I think the team, like most of us are too young to have that really on our radar as a major concern, right? (Investigator 03)

As researchers engaged with people who are ultimately the target of health services and interventions, they felt they were able to collaboratively design more relevant and meaningful interventions. It provided an opportunity to “get out of our academic brains” (Staff 05) and understand intervention concepts and designs from patients’ perspectives.

### Impacts on Researchers

Researchers highlighted two ways that consulting with patients impacted them on personal and interpersonal levels: developing communication skills and increased job satisfaction.

#### Better Communication

Researchers reported consulting with patients improved their ability to communicate research in lay terms; specifically, it led researchers to think carefully about how to present information and ask for input using non-technical language. Researchers believed this effort would benefit them when they interacted with study participants and eventually to disseminate their research more broadly: “…it gives us a better understanding of how we can talk about the study in an approachable way but also like what parts of the study may resonate more with [patients]” (Staff 01). An investigator noted that focusing on clearly discussing scientific topics with patient consultants helped the entire team communicate more clearly with each other.

I had to be mindful about preparing at least for these meetings where we were presenting information to the Veterans. I think sometimes it feels like, okay, I’m having to take more time to explain something but I think in reality we have these like multidisciplinary teams where not everyone understands the statistics. So I think it clarifies things for everybody on the team. (Investigator 03)

#### Enhanced Job Satisfaction

Researchers appreciated working closely with patients, and some specifically noted that the experience was rewarding and satisfying. Although typically only one or two patient consultants were involved in a given project, their inclusion helped researchers to feel more connected to VA’s overall mission and to the patient population whose outcomes they aimed to improve through research.

Like it just makes me feel a little bit more connected to the mission. I mean we’ve always known that the goal is to improve health services for Veterans. But I think having them as part of the team that’s helping you do that, you know, at least makes you feel like well there’s at least a couple Veterans who maybe appreciate what I’m doing even if [the project results don’t get immediate uptake clinically]. (Investigator 03)

Researchers also found it gratifying to know consultants personally benefitted from engagement. Enhanced job satisfaction was particularly pronounced for research staff, with patient engagement seen as a “bright spot” in their day. As one project manager noted, staff are often focused on administrative practicalities while principal investigators have “a theory and the grand ideas”; working together with patients to improve interventions was “energizing” (Staff 05).

### Impacts on Patient Consultants

Patient consultants highlighted a range of benefits from being involved on research teams. We categorized these benefits into two domains: personal satisfaction and appreciation for research.

#### Personal Satisfaction

Patients largely reported positive experiences working on research teams, including feelings of satisfaction and purpose. Patient consultants found it gratifying to see how their involvement in research contributed to a grant or research study and appreciated knowing their ideas were implemented.

So it may be exciting to see, you know, the fruits of the labor as you say. What has came about with all these different people with all these little specialties and expertise and different parts making something come together… I got to see some of the things that I had brought up get implemented and put in. So it’s like, I felt like I played a part. (Patient 03)

Receiving specific, positive feedback from researchers about how their input contributed to the project enhanced patient consultants’ sense of satisfaction from engaging. Conversely, this benefit was attenuated when there was poor post-engagement follow-up about the status of the project or the utility of consultants’ feedback. When asked if they were able to achieve their goal of giving back to others, Patient 05 replied: “[maybe] to some degree but I never heard back… for all I know, they’re in the research phase or publication phase or it never got funded.”

Participating in the VCN could also contribute to feeling a sense of purpose from helping other patients. For patient consultants who overcame personal difficulties (e.g., health issues, homelessness, incarceration), participating in research was especially rewarding when they were able to give back to others in similar situations and reinforce how much they had grown.

[Being on a research team] kind of makes some of my experiences more worthwhile. They’ve got some meaning behind them because a lot of what I put myself through isn’t what a lot of people would consider positive experiences. But if they end up helping somebody else down the road then that turns it into a positive. (Patient 11)

#### Awareness of and Appreciation for Research

Patient consultants learned about the research process and better appreciated the value of patient engagement in research. Participating let patient consultants see how VA is trying to improve its’ health services, for which many expressed appreciation:

Becoming involved here at [CHOIR] has cleared up, has made me realize that VA is working to improve things. They are trying to make things better. Amongst many Veterans “the consensus is the VA doesn’t give a shit.” But knowing what you do over here in “the secret squirrel land” let’s me know the VA is trying to improve. Whereas before I when I was going through stuff, I thought “why wouldn’t they fix this?” (Patient09)

Through their engagement, patients had more insight into how the research process works within the VA, including how groups work at multiple levels of the healthcare system to help improve care. Increased exposure to the research process, however, did lead some patient consultants to perceive research as inefficient or bureaucratic (for example, the long review times for grants and Institutional Review Board applications).

### Updated Logic Model

**Figure 3:**
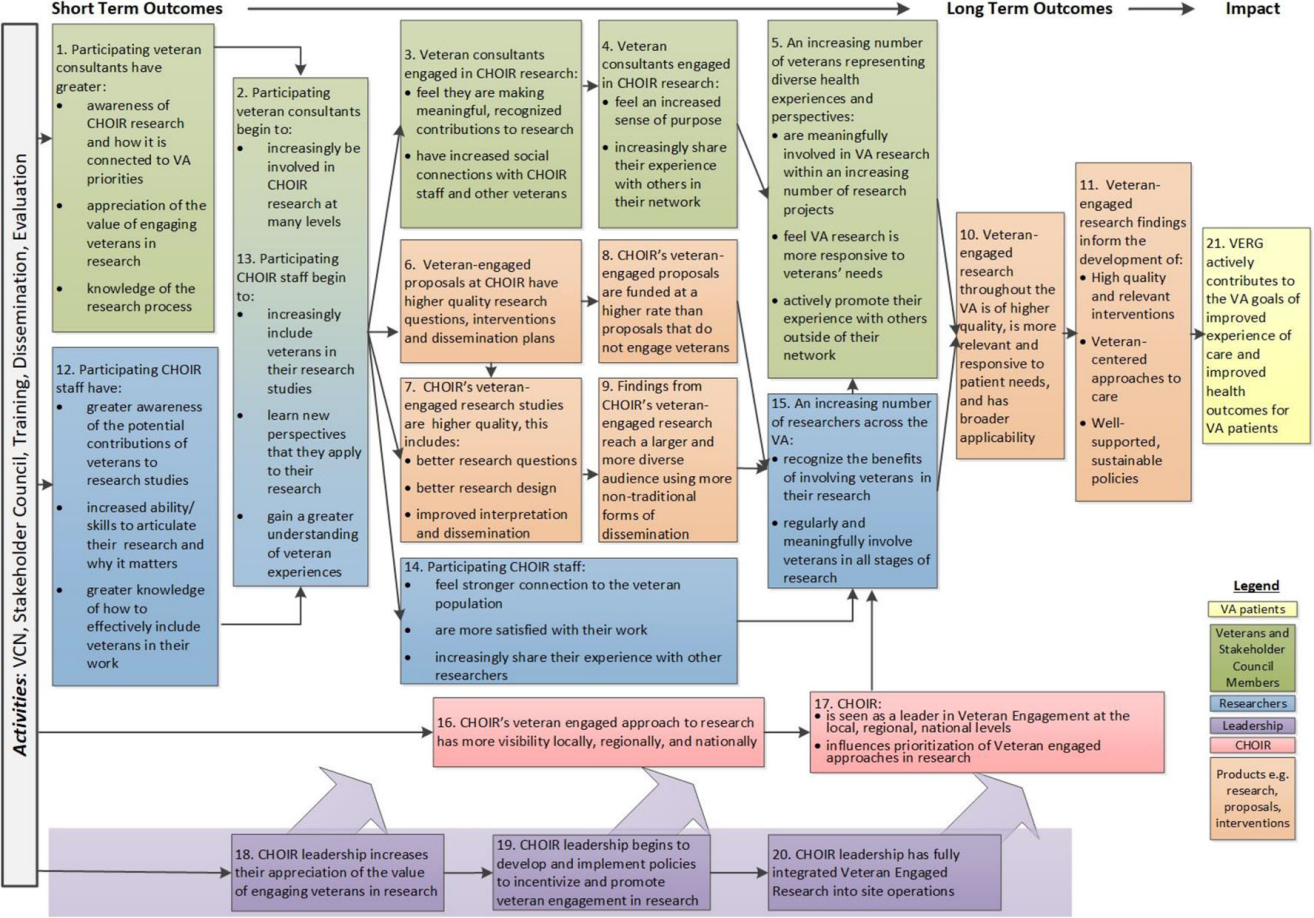
Updated full logic model for veteran engagement at CHOIR. Anticipated short-, medium-, and long-term outcomes of Veteran engagement in research at the Center for Healthcare Organization and Implementation Research (CHOIR), including hypothesized outcomes for Veterans engaged in research (green boxes 1–5), researchers (blue boxes 12–-15), research studies and resulting evidence-based interventions (orange boxes 6–11), the research center (red boxes 16–17), research center leadership (purple boxes 18–20), and Department of Veterans Affairs (VA) patients at large (yellow, box 21).

Finally, we compared findings to our logic model to reflect on unobserved outcomes. While we initially expected patient consultants to gain research skills, this usually did not occur. Instead, patient consultants shared their lived experiences with and gave general feedback to researchers, who then incorporated these insights into research products (rather than asking consultants to learn how to perform research directly). Despite not gaining research skills, patients were still able to make meaningful, recognized contributions to research from which they derived satisfaction and purpose. Thus, the outcome did not seem necessary to achieving long-term impact; it was removed from the logic model. Patients also largely reported not talking about research or their consulting experiences with other Veterans; researchers did not discuss whether or not they shared their engagement experiences with other researchers. While not observed in this evaluation, we felt these outcomes were still potentially achievable and important to developing a larger culture of patient engagement within our medical center and enhancing our center’s reputation as a leader in patient engagement. As such, they were retained in the logic model. Based on these and our prior findings, we updated our logic model to reflect our current understanding of the mechanisms of impact for our patient engagement program (Fig. 3).

## DISCUSSION

There are few examples in the literature that demonstrate both the methods for and value of evaluating the influence of engaging patients as partners in the research process. Creating a logic model compels researchers to explicitly articulate their values, assumptions, and goals regarding patient engagement.^34, 35^ Program evaluations can then assess alignment between logic models and reality, reflect on what outcome pathways are critical for achieving desired impacts, and adapt program activities and/or outcome expectations accordingly.^24, 36^

Our logic model presents how patient engagement led to perceived improvements in the quality of research grants, interventions, and study materials across multiple VA health services research projects, alongside benefits to researchers and patient consultants. Our evaluation process also prompted us to assess reasons for and the importance of unobserved outcomes. For example, we learned that patient consultants largely did not gain research skills, calling attention to our center’s lack of research skill development activities (such as training on research methods) while simultaneously clarifying that this outcome did not seem to be meaningful to program users or necessary to achieve long-term impact. Finally, the evaluation highlighted needed process improvements to enhance program outcomes, such as the need for research teams to more proactively and consistently communicate about research timelines and the results of patient consultants’ contributions.

Comparing logic models across programs can illuminate shared goals and values inherent to patient-oriented research.^21, 37^ Similar to our program, other logic models have also highlighted patient consultants’ sense of empowerment, researchers’ improved understanding of patients’ perspectives, and more relevant and feasible research studies.^15, 18^ Comparing patient engagement programs can also help highlight common methods for and impacts of patient engagement.^5^ However, additional cross-organization comparative research is needed to explore how institutional context, group dynamics, and personal relationships influence the outcomes of patient engagement.^38^

### Limitations

This evaluation focused on assessing potential impacts of patient engagement in one research center; findings may not be applicable to other patient engagement initiatives. Because of the timing of this evaluation, our interview sample reflects mostly early adopters of patient engagement. Continued monitoring is necessary to understand whether late adopters experience the same benefits from participation, or if additional facilitation is needed to sustain engagement quality. In addition, interviewees’ comments largely reflect perceived impacts on research proposals and studies. Tracking projects longitudinally through data analysis and dissemination will be necessary to see if observed outcomes lead to desired long-term impacts. Future research verifying perceived impacts with third parties (such as grant reviewers and study participants) may be helpful in demonstrating the links between patient engagement and improved grants, interventions, and study materials.

While our findings on the benefits of patient engagement are important confirmation of and expansion to prior literature,^15, 39^ what we believe is more universally valuable for a broad array of patient engagement programs is the evaluation process described herein. The process of creating a logic model for a patient engagement program can help other research centers articulate the relationship between their goals, activities, outcomes, and impacts; evaluations guided by logic models can help research centers investigate their assumptions about the value and impact of patent engagement activities. This process can help organizations reconsider whether unobserved short-term outcomes are vital to program success and increase support towards those deemed critical. Particularly when patient engagement is not financially supported,^40^ this process can help organizations prioritize their patient engagement approach and resources.^40^ Ultimately, these tailored reflective and evaluative practices can help foster a culture of continuous organizational learning around patient engagement that best supports the development of meaningful, relevant health research.

## Supplementary Information

ESM 1 (PDF 120 kb)

## Declarations

### Disclaimer

The contents of this manuscript do not represent the views of the US Department of Veterans Affairs or the United States Government.
