# Supplementary material for: Evaluating the Impacts of Patient Engagement on Health Services Research Teams: Lessons from the Veteran Consulting Network
Source: J Gen Intern Med. 2022 Mar 29;37(Suppl 1):33–41. doi: 10.1007/s11606-021-06987-z (PMC8993982; doi:10.1007/s11606-021-06987-z)
Supplement: Supplementary file 1 — (PDF 120 kb) [file 11606_2021_6987_MOESM1_ESM.pdf]

Appendix 1. Interview Guides

Interview Guide for Veteran Consultants

1. Can you start out by telling me a little bit about the research project(s) you were involved with?
   - a. What kinds of activities have you participated in?
   - b. What has the experience of being on a research team been like for you?

2. How did you come to be a consultant on this project(s)?

3. Thinking back, can you remember why you originally wanted to participate on a research team?
   - a. Have you been able to achieve any of these goals yet?
   - b. In general, has your experience measured up to your initial expectations for doing this work?
     - b1. [If not going well . . .] What would have to change to improve your experience and help you achieve your goals for being a Veteran consultant?
     - b2. [If going well . . .] Can you give me an example of what helped you feel that way?

4. Have there been any other benefits from being a Veteran consultant you didn't expect? (Probe: sense of purpose, knowledge of research process, research skills)

5. Do you feel like you were able to contribute to this research project?
   - a. Can you give me an example of what you did and how it made a difference?
     - [For example, helping researchers understand what information patients need, helping design a survey, analyzing or reviewing results, etc.]
   - b. Do you feel like your contributions were valued by the team? What made you feel like that?

6. What things have supported your ongoing involvement on a research team?
   - a. What are the top 1 or 2 things that members of your research team did to support your involvement?
   - b. Is there anything the Veteran Consultant Network did that helped you be involved in research? (Probes: orientation, payment)

7. Now I'd like to ask you about any challenges or barriers you faced as a consultant on this project. Was there anything that got in the way of your involvement? (Probes: unsure of role, input not valued, time commitment, scheduling, transportation, etc.)
   - a. Why do you think you faced these challenges?
   - b. What could have helped you with these challenges?
   - c. Are there any changes VA could make to better support other Veteran consultants? (Probes: different research roles/tasks, higher payment/expense reimbursement, orientation/training, opportunities for peer networking, more frequent communication, etc.)

8. How has working on a research team affected how you feel about VA, if it has affected it at all? (Probes: trust in VA, perception VA is responsive to Veteran needs, positive communications to other Veterans about VA, etc.)

9. What do you think would be the best way to communicate with Veterans and their families and caregivers about the research VA is doing?

10. Do you have any thoughts on how we can work to keep Veteran consultants involved while they are waiting to be matched to a project or while they are waiting for a project to be funded?

11. Is there anything I haven't asked about, or we haven't discussed, that you feel would be important for us to know about yours or other Veterans' involvement in research?

<u>Interview Guide for Researchers</u>

1. Can you start out by telling me a little bit about the research project(s) you worked on with Veteran consultants?
    a. How did you come to have a Veteran consultant on your project(s)?
    b. Which Veteran consultant(s) did you work with?
    c. What has the experience of having a Veteran consultant on your research team been like for you and your team?

2. Thinking back, can you remember what you originally expected working with a Veteran consultant to be like?
    a. How has your experience measured up to those initial expectations?
    b. [If they say it is meeting expectations…] Can you give an example of that?
    b. [If they say it is not going as well as expected…] What would have to change to make working with a Veteran consultant be what you hoped?

3. What kinds of activities did your Veteran consultant(s) participate in?

4. Do you feel like you like the Veteran consultant(s) you worked with was/were able to contribute to your research project(s)?
    a. Can you give me some examples of what they did and how it made a difference?

5. Beyond this specific project, have there been any benefits of having Veteran consultants on you or your research team?
    a. How has this experience affected your research? (Probe: new research perspectives)
    b. How has this experience benefited you personally, if at all? (Probe: awareness of and ability to do Veteran engagement; improved ability to communicate about research topic)
    c. Have there been any benefits you did not expect?

6. How has working on with Veteran consultant(s) affected how you feel about Veterans and the VA, if it has affected it at all? (Probe: stronger connection to Veteran population, job satisfaction)

7. What things have supported your ability to involve Veteran consultants in your research?

a. What are the top 1 or 2 things that you or members of your research team did to support your Veteran consultant's involvement?

b. Is there anything that VERG, CHOIR, or VA has done that helped you involve Veteran consultants more effectively?

8. Now I'd like to ask you about any challenges or barriers you faced when including consultants on your project(s). Was there anything that got in the way of involving your consultant? (Probes: unsure of their role, scheduling issues, payment, etc.)

a. Why do you think your consultant or your team faced these challenges?

b. What could have helped you with these challenges?

c. Are there any changes CHOIR or VA could make to better support other Veteran consultants? (Probes: different research roles/tasks, higher payment/expense reimbursement, orientation/training, opportunities for peer networking, more frequent communication, etc.)

9. How likely are you to include Veteran consultants on your team going forward? What would you do differently on your next research project?

10. Do you have any tips for other investigators/staff members for working with Veteran consultants?

11. Is there anything I haven't asked about, or we haven't discussed, that you feel would be important for us to know about involving Veteran consultants in research?
